# Arenavirus Variations Due to Host-Specific Adaptation

**DOI:** 10.3390/v5010241

**Published:** 2013-01-17

**Authors:** Juan C. Zapata, Maria S. Salvato

**Affiliations:** Institute of Human Virology-School of Medicine, University of Maryland, Baltimore, MD, 21201, USA; E-Mail: jczapata@ihv.umaryland.edu

**Keywords:** arenavirus, LCMV-Clone13, LCMV-Armstrong, Mopeia/Lassa reassortant ML29, mutation, viral population, co-evolution

## Abstract

Arenavirus particles are enveloped and contain two single-strand RNA genomic segments with ambisense coding. Genetic plasticity of the arenaviruses comes from transcription errors, segment reassortment, and permissive genomic packaging, and results in their remarkable ability, as a group, to infect a wide variety of hosts. In this review, we discuss some *in vitro* studies of virus genetic and phenotypic variation after exposure to selective pressures such as high viral dose, mutagens and antivirals. Additionally, we discuss the variation *in vivo* of selected isolates of Old World arenaviruses, particularly after infection of different animal species. We also discuss the recent emergence of new arenaviruses in the context of our observations of sequence variations that appear to be host-specific.

## 1. Background

Here we review genetic and phenotypic variations of arenaviruses at the level of virus families, at the level of genera, at the level of species, and finally within those entities known as “strains” or “isolates”. Sequence analysis reveals that the *Arenaviridae*, the *Filoviridae* and the *Bunyaviridae* share some structural motifs that could be derived from common ancestry or functions. Classical studies on the prototypic arenavirus species, lymphocytic choriomeningitis virus (LCMV), have followed the sequence and phenotypic variation of the LCMV-Clone 13 and LCMV-Armstrong strains *in vivo* and *in vitro*. Such studies shed light on our own analyses of the Mopeia/Lassa reassortant virus, ML29 as it varies after passage through a variety of host species.

### 1.1. Arenavirus Structure

**Figure 1:**
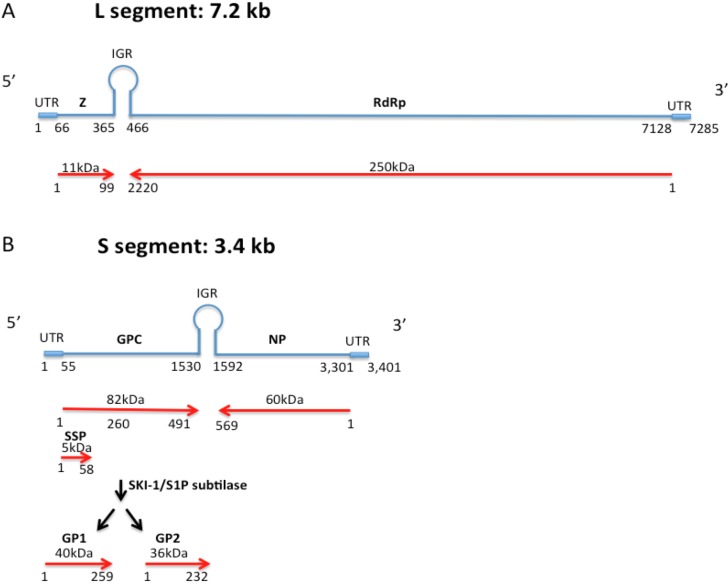
Arenavirus genome structure from 5’ to 3’ end. **A**) The LASV L genome segment (7.2 kb) is represented in blue and is composed of an untranslated region (UTR) from nucleotide 1 to 66 and from 7129 to 7285, the gene encoding the zinc-binding protein (Z) from nucleotide 67 to 365, the intergenic region IGR from nucleotide 365 to 466, and the RNA-dependent RNA polymerase protein encoding gene (RdRp) from nucleotide 466 to 7128. **B**) The LASV S segment genome (blue lines) contains the untranslated region (UTR) from nucleotide 1 to 55 and from 3302 to 3401, the gene encoding the glycoprotein precursor protein (GPC) from nucleotide 57 to 1530, the intergenic region IGR from nucleotide 1531 to 1592, and the nucleocapsid protein encoding gene (NP) from nucleotide 1593 to 3301. The red arrows represent the 491 amino acid long GPC with its stable signal peptide (SSP) and glycoproteins 1 and 2 (GP1 and GP2) produced after maturation cleavage. The 569 amino acid long NP is shown encoded in the antisense orientation.

**Figure 2:**
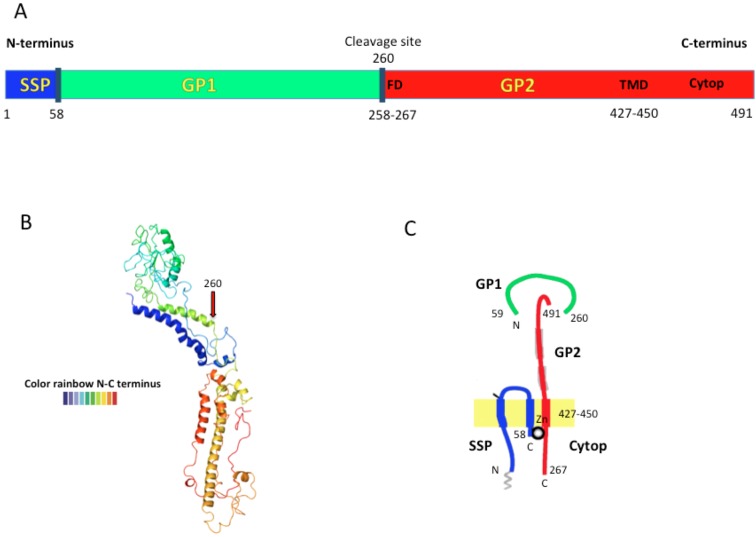
The LASV envelope glycoprotein precursor (GPC) structure is shown in **A**) from N- to C-terminus containing SSP, GP1 and GP2 proteins. The dark blue lines represent the cleavage points. The fusion domain (FD); transmembrane domain (TMD), and cytoplasmic domain (Cytop) are shown in brown letters (modified from [19]). **B**) The predicted Lassa-Josiah GPC structure obtained by open-source software (Phyre^2^). The structure goes from N-terminus (blue) to C-terminus (red). **C**) Schematic representation of the trimeric GPC subunit assembled in the cell membrane. GP1 is the most external protein bound to GP2 that is embedded in the lipid membrane. GP2 is thought to interact with SSP through an inter-subunit zinc finger (ball) (modified from [20]).

Arenaviruses have enveloped particles containing bi-segmented genomes of single-stranded RNA encoding four viral proteins in an ambisense manner. The small segment called S (~3.4kb), encodes the glycoprotein precursor (GPC) and the nucleocapsid protein (NP) that are the most important immunogens of the virus (Figure 1 and Figure 2). The NP and GPC sequences are separated by a noncoding intergenic region (IGR) [1]. The GPC is processed into a signal peptide (SSP), and into portions GP1 and GP2 (Figure 1 and Figure 2), which function to mediate viral assembly, entry, and uncoating and to determine cell tropism. NP has multiple functions: it encapsidates the arenavirus genome segments, interacts with L protein to form the RNP core for RNA replication and transcription, associates with Z protein for viral assembly, plays an important role in suppressing the innate immune response, and has exonuclease and nucleotide binding activity [2,3,4,5,6,7,8,9].

The Large (L) segment (~7.2Kb), encodes the small zinc-binding protein (Z) that functions as a matrix protein, interacts with L and NP and other host proteins, plays a role in viral transcription and replication, has pro-apoptotic activity and is essential for virus budding [3,5,10,11,12,13,14]. The L segment also encodes the L protein that is an RNA-dependent RNA polymerase (RdRp). L and NP are the minimal *trans*-acting virus factors required for replication and transcription [2,15,16]. L and Z are also separated by an IGR [17]. Both RNA segments are flanked by noncoding regions (UTR) that function with the IGR as *cis*-acting elements for RNA replication and transcription [15,17,18] (Figure 1).

Arenavirus molecular biology so far has been focused on characterizing the role of each domain of the viral proteins *in vitro* using reverse genetics platforms. However, since single mutations can affect the functions of several proteins in a complex, an additional approach has been to isolate viruses with different biological properties *in vivo*, and then to determine which mutations contribute most to the functions related to the *in vivo* phenotype. New molecular and crystal structures now elucidate the role of each viral component and guide the production of better vaccine candidates and antiviral compounds.

### 1.2. Arenavirus Taxonomy

The International Committee on Taxonomy of Viruses (ICTV) established the family *Arenaviridae*, based on morphological, physiochemical, and serological parameters [21,22]. This family now includes 25 species of viruses (insider update by MSS, a member of the International Committee on Taxonomy of Viruses or ICTV-arenavirus group). All the currently-classified arenaviruses are carried by rodents, except for Tacaribe virus that is carried by bats (see virus abbreviations in Supplementary Table 1). At least 10 of these viruses occasionally infect human beings causing zoonotic diseases [22,23]. The arenaviruses can be divided into two serocomplexes: The Old World (OW) and the New World (NW), also known as the LASV-LCMV complex and the Tacaribe complex, respectively. The OW group includes the globally-distributed LCMV and the African arenaviruses that infect rodents of the family *Muridae*, subfamily *Murinae*. The NW viruses include South and North American arenaviruses infecting rodents of the family *Muridae*, subfamily *Sigmodontinae* [24]. It is highly possible that long-term co-evolution with the Sigmodontine rodents drove the evolution of distinct New World viral species [25].

In addition to the criteria that divide the *Arenaviridae* into NW and OW groups, species demarcations are determined by the following criteria of their member viruses: significant differences in antigenic cross-reactivity and cross-neutralization; role as an etiological agent of disease (or not) in humans; presence in a defined geographical area, host species or group of species, and significant protein sequence differences compared to other viruses in the genus (*i.e.,* showing a divergence between viruses of different species of at least 12% in the nucleoprotein sequence). However, there are still some poorly-defined criteria for classification. For example, consider the criterion that the amino acid sequence of the NP has less than 88% homology to the closest arenavirus. It is interesting that some Lassa isolates vary more than 12% from each other, and should be classified in different species but they are not. Another critical point is the possibility of reassortment among viruses assigned to the same species. It is odd that Lassa and Mopeia were able to reassort, even though they are assigned to different species, perhaps due to compatibility between the Lassa and Mopeia terminal sequences. In addition, there are several reports of arenaviruses detected by molecular techniques from human or animal samples, however some of them are not yet associated with human diseases or failed to replicate in cell cultures (see Supplementary Table 1). With non-isolated viruses, it is impossible to study serological and morphological parameters to give an accurate characterization. Although, they can not be classified as new Arenavirus species until infectious isolates become available [26] (Supplementary Table 1), it is important to capture and record their characteristics because they shed light on the evolution of the arenaviruses.

### 1.3. Antigenic Characteristics

Broad serological cross-reaction and neutralizing or complement-fixing antibodies are used to identify the OW and NW arenavirus complexes [27,28,29,30,31,32]. Monoclonal antibodies (MAbs) produced against the GP2 of two African arenaviruses reacted broadly against American arenaviruses, demonstrating highly conserved epitopes in this family [33,34]. In a more narrow way, Mabs against JUNV NP reacted only with NW arenaviruses [35] or reacted only with isolates from local foci, suggesting a strong antigenic stability of those viruses in local areas [34]. Despite those results, several attempts failed to define clearer serological differences between arenaviruses useful for species classification [34,36]. However, the close antigenic relationship could be useful to design immuno-prevention systems to induce cross-protection against related strains. For example, guinea pigs and marmosets inoculated with TCRV are protected against JUNV disease [23,37]. Candid #1, a vaccine for JUNV, cross-protected rhesus monkeys from disease after challenge with MACV; both viruses belong to clade B of NW viruses [38]. Similarly, guinea pigs, marmosets or rhesus monkeys are protected from LASV disease after inoculation with MOPV and endemic regions for this virus have no reported Lassa Fever (LF) cases even though LASV and MOPV share the same rodent host [39,40]. In conclusion, the antigenic relationships between members of the family *Arenaviridae* suggests that viruses assigned to a given species are very stable in small geographical areas, and that OW and NW groups diverged a long time ago.

### 1.4. Virus-Host Coevolution

Arenaviruses normally produce persistent infections in rodents, with chronic viremia and viruria, spreading virus through urine, feces, and saliva to other rodents and accidentally infecting humans or other mammals [41,42,43]. Although it is generally thought that infections in rodents are asymptomatic, some studies showed that infection can cause weight changes, widespread viral dissemination, increased mortality, and reduction in size and fecundity of survivors. All those changes depended on viral loads, viral genomic sequences, and the genetic backgrounds of the mice [44,45,46]. A strong example of arenavirus infection affecting mouse breeding are the experimental infections of large vesper mouse (*Calomys callosus*) females with MACV resulting in female sterility [47]. In other studies, adult white-throated wood rat (*Neotoma albigula*) pregnant females were seronegative for WWAV in a population with 60% seropositivity, indicating that little vertical transmission occurred. Similar observations are reported for the drylands vesper mouse (*Calomys musculinus*)-Junin virus disease model, where transmission is more common between adult males and associated with the presence of scars, implying violent transmission during aggressive encounters [48,49,50]. In contrast, the infection of rodents classified as *Mastomys sp.* with LASV seems to have no sex or age bias suggesting more frequent vertical transmission from dam to pups [51]. From this evidence, it can be inferred that vertical transmission has a greater impact in reducing mouse populations than horizontal transmission; exerting strong selective pressures on mice infected early in life. 

In general, diseases produced by arenaviruses are located in endemic foci determined by the presence of the natural host. However, the geographic localization of a specific arenavirus can be more restricted than the distribution of its reservoir. LCMV is an exception with worldwide distribution through common house mice from the family *Muridae*, or hamsters from the family *Cricetidae*. African viruses circulate in endemic areas between rodents from the family *Muridae*, genus Mastomys, Praomys, or Arvicanthis. The American arenaviruses circulate in specific areas between rodents from the family *Cricetidae,* genera *Oryzomys, Sigmodon, Neotoma, Nephelomys, Oecomys, Calomys, Zygodontomys, Neacomys*, or *Akodon* (Supplementary Table 1). TACV is an exception believed to circulate in bats (*Artibeus sp.*) (Supplementary Table 1). The fact that each arenavirus is usually associated with a single type of rodent or closely related rodents [25], together with phylogenetic studies in both virus and host, indicates an apparent codivergence between them [52]. The current rodent radiation hypothesis [53] states that holoartic cricetids (ancestor of murids) spread to the Americas from North to South in different waves evolving into ancestors of the *Neotominae* and *Sigmodontinae* sub-families, probably carrying the NW arenavirus ancestors. Additionally, cricetids spread to the Asian continent following different migration waves; from there, they continued to Europe and then to North Africa evolving toward the *Muridae* sub-family and carrying the OW arenavirus ancestors. The sub-Saharan conditions of radiation, weather, human migration, influenced the high diversification and isolation of those rodents that populated South Africa and carried the OW African arenaviruses [53]. In the case of the newly discovered Lujo virus, the sequence analysis showed that it is most strongly related to OW viruses but shares GP sequences with the NW viruses [54]. Similarly, the characteristics of other OW viruses coalesce into distinct species, suggesting that, within the African continent, signatures of co-evolution might have been obliterated by multiple evolutionary events in which viruses jumped from one host to another [55,56]. 

Arenavirus-rodent interactions exemplify virus-host codivergence, in which one rodent species diverges from another so much, that the viruses of each host are no longer able to infect the same hosts equally well. Codivergence and cospeciation are similar: if two species are closely associated, as in viruses and their hosts, they might speciate or evolve in parallel. Thus it could eventually be possible to assign host species by the population of parasites (including viruses) they carry [52]. An alternative hypothesis suggests a combination of co-speciation events and virus transfer among rodent species that may explain the codivergence incongruities seen in some members of this family [57,58]. One study in NW arenaviruses found that both events occurred during arenaviruses evolution suggesting not only codivergence but also evolution via host switching, implying an arenaviral potential for becoming panzootic [59].

Ten arenaviruses are known to cause natural (LCMV, LASV, LUJV, MACV, JUNV, GTOV, CHPV, and WWAV) or laboratory acquired (TCRV, FLEV, and SABV) human disease in specific geographical regions (Supplementary Table 1). Those viruses do not appear to be monophyletic, suggesting that the pathogenic phenotype has risen in multiple independent events during virus evolution [57].

There is also evidence of selective pressures on human populations by arenaviruses. Two genes related to LASV infectivity and immunity (LARGE and interleukin 21) had been associated with a possible positive selection after viral infection leading to LF resistance. Those genetic traits are evidence that the human incidental host co-evolved with LASV. In addition, LASV infection in pregnant women results in nearly 100% fetal lethality exerting a strong selective pressure in the endemic regions. Thus, arenaviruses exert selective pressures on both human and carrier host populations [60,61].

During the codivergence process, OW and NW arenaviruses evolved to use different cell receptors (alpha-dystroglycan *vs.* transferrin receptor 1), different entry mechanism (delivery to endosomes via vesicular trafficking *vs.* endocytosis), and different protective immune responses in rodent and in primate incidental hosts (innate *vs.* acquired immunity) [62,63,64,65,66,67,68]. All those co-evolution findings raise interesting questions such as: Why are arenaviruses so species-specific despite high mutation rates that could increase host range? What mouse or monkey genes are controlling resistance to arenavirus infections? Did non-pathogenic arenaviruses and mice or human populations co-evolve to co-exist without disease? Those answers could help us understand arenavirus co-divergence and identify target genes for disease treatment.

## 2. Arenavirus Plasticity

The arenaviruses, like other RNA viruses, are highly divergent due to high mutation rates from a low-fidelity viral RdRp and due to reassortment and possibly recombination events that contributed to viral diversification during arenavirus evolution [25,69,70]. Although some reassortants were produced *in vitro*, those experiments indicated that there are restrictions that prevent recovery of all possible combinations [71,72,73,74,75,76]. So far, no reassortant arenavirus has been isolated from nature and recombination appears to be rare and occurs only between phylogenetically close strains [52,70,77,78]. Therefore, the high frequency of transcription errors appears to be the main driver of arenavirus evolution. The estimated mutation rate of RNA viruses ranges from 10^-3^ to 10^-5^ per nucleotide incorporated during replication [79,80]. Lethal mutagenesis studies *in vitro* estimate that the error frequencies of the LCMV RdRp are between 1 × 10^-4^ and 5.7 × 10^-4^ substitutions per nucleotide [81,82]. Those results are consistent with the genetic heterogeneity seen in other arenaviruses [83]. Comparison of nucleic acid and protein sequences within specific arenaviruses showed identities ranging from 90–95% [84], even if they were isolated in the same region [85,86]; and viruses isolated from different regions ranged from 78 to 86% [85,86]. Sequence comparison of all proteins of seven pathogenic arenaviruses showed identities ranging from 44 to 63% [84]. LASV isolate variation is the highest among this family with nucleotide and amino acid variations of 27% and 15% respectively, followed by PIRV with 26% and 16% variations isolated in very close geographical regions [87,88]. The genetic diversity within and between isolated arenavirus groups, suggests that the spatial heterogeneity may be reflected in host range and pathogenicity. Consequently, sequence analysis of new virus isolates could be useful for tracking the source of arenaviruses outbreaks [86].

### 2.1 Arenavirus Variation *in vitro*

When referring to a virus strain we are describing the most abundant variant from a closely-related virus swarm containing individual particles with broadly-distributed mutations. A viral isolate containing many variants, called quasi-species, could act as a unit of selection through a continuous dynamic process of genetic variation, competition, and selection [89]. However, it is important to emphasize that the whole “virus swarm” contributes to the characteristics of the virus strain and will be the target of selection instead of individual variants. For example, some variants carrying lethal mutations not only compete with the fittest replicating unit but also cooperate with other mutants complementing each other, thus assuring the survival of the units containing "lethal mutations" [90]. Several studies describe the effect of the mutant spectra in the virus population phenotype making it more virulent or more attenuated according to the predominant quasi-species. For example, defective interfering particles could play a role in attenuating the virulence of a viral swarm by interfering with virus replication [91,92,93,94,95]. Therefore it is important to take into account that serial passages of arenaviruses in cell lines or animals could lead to the accumulation of mutations changing the viral phenotype from the natural reservoirs or from clinical samples [14,84,96]. In fact, consecutive passage of arenavirus through cell lines has been used to detect viral strains with pathogenicity differing from the original viral stock [72,97,98]. However, *in vitro* changes should occur at a different rate than those seen *in vivo,* due to the absence of several selective forces (e.g., immune system, a variety of receptors, different tissue compartments).

Several studies explore arenavirus variability under specific selective pressures as a model of viral evolution. For instance, serial passages of LCMV in the presence of a mutagen such as 5-fluorouracil (5-FU), and an anti-viral like ribavirin leads the viral population into extinction in an event known as “error catastrophe” or “lethal mutagenesis” [81,99,100]. This phenomenon is explained as enhanced mutation rates that produce accumulation of many non-viable mutants resulting in increased sensitivity to anti-virals and an abortive infection. In other words, genome flexibility allows the adaptation of viral populations to environmental changes; however there is an “error threshold” beyond which viral fitness is considerably reduced and the virus population may face “viral extinction” [83,101,102]. On the other hand, with low genomic variation (increased fidelity of replication) the viral population does not have the capacity to adapt to new conditions due to low quasi-species diversity. Under those circumstances, viral survival can also be compromised [103,104].

Cell culture manipulations of Lassa and Mopeia viruses revealed that these African viruses species were so closely related as to be able to reassort with one another [72]. Initially, Vero cell cultures were co-infected with a Lassa strain (Josiah) and a Mopeia strain (AN20410). Both small plaque (Mopeia phenotype) and large plaque (Lassa phenotype) progeny viruses were observed and some proved to be reassortants between Lassa and Mopeia [72]. The progeny isolate that grew best, ML29, was a small-plaque isolate with its large genomic segment (L) from Mopeia and its small segment from Lassa [105]. This reassortant had 18 mutations with respect to the parental viruses [106]. These mutations included 3 amino acid changes (Mopeia to ML29) in the non-conserved regions of the polymerase (Y851N, R1233G and D2136N), one amino acid change in GP2 (Lassa to ML29; K272E), and two amino acid changes in NP (A485D within the DEDD exonuclease of the NP C-terminus [9], and N173S). Two mutations in the panhandle (complementary termini) region and several mutations that did not impact the proteome were hypothesized to stabilize the RNA structure of the reassortant. Since ML29 is a candidate attenuated vaccine for Lassa fever, the stability of its phenotype and genome were assessed after 12 passages in Vero cell culture. The small plaque phenotype and the ability to protect mice from lethal intracerebral inoculation was retained through all 12 passages, as was the consensus sequence of the open reading frames. However, deep sequence analysis revealed the gradual increase in single nucleotide polymorphisms (SNPs) with *in vitro* passage, though none of the SNPs exceeded 25% frequency within the viral population [107]. This agrees with the idea that one viral preparation can maintain a swarm of quasi-species with a variety of sequences; yet retain its phenotypic characteristics [102].

Thus, changes in virus diversity *in vitro*, increasing or decreasing mutation rates, can affect virus fitness and viral tropism and produce a spectrum of outcomes depending on particular conditions of the virus-host interaction. Those findings were explored with the goal of using “lethal mutagenesis” as an anti-viral treatment, and viral clones with high fidelity polymerases as attenuated vaccines [103,108].

### 2.2. Arenavirus Variation within a Single Host

Chronic infections are the best models for studying the structure and evolution of a virus population within a single host since viruses have a chance to evolve after extensive replication, genomic changes and selection. The prototypic virus LCMV is able to produce acute or persistent infections in mice and has been used as a model to study viral population dynamics *in vivo* [109,110]. The intra-host viral variability and the existence of tissue-specific virus populations had been studied in plant and mammalian hosts, suggesting that viral compartmentalization is caused by selective pressure exerted by each target tissue [109,111,112,113,114,115,116,117,118,119,120].

During the peak of an arenavirus acute infection in monkeys, the number of viral particles in blood is around 1 × 10^7^ PFU/ml [39,121,122,123]. With mutation rates approximately 1 × 10^-4^ substitutions per nucleotide and a virus of approximately 10^4^ nucleotides in size, by the time maximum viremia is reached (after several days of replication) every possible mutation had multiple chances to occur. In fact there is a close correlation between arenavirus concentration and severity of disease. Analysis of cytotoxic T lymphocyte or neutralizing antibody escape mutants in mice, showed mutation rates around 3 × 10^-4^ substitutions per nucleotide and suggested that those mutations are present and are selected from the virus stock instead of appearing spontaneously [124,125,126]. This process is limited and guided by the selective pressure exerted by the host and its different conditions. For instance, laboratory mice infected at the time of birth with LCMV-Armstrong develop persistent infection, and after 8–6 weeks, virus isolated from the nervous system (CNS) was similar to the parental virus, causing acute infections and strong immune responses in adult mice; whereas isolates from spleen (such as LCMV-Docile and LCMV-Clone 13) produced chronic infections with suppressed T-cell responses and susceptibility to opportunistic infections [109,110,127,128].

**Figure 3:**
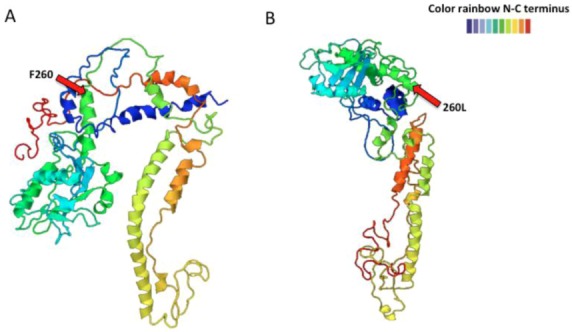
LCMV-ARM53b (**A**) and -Clone 13 GPC (**B**) predicted structure using the Phyre^2^ program. One amino acid change from F to L at position 260 alters the predicted GPC structure. The structure goes from N-terminus in blue color to the C-terminus in red color.

Sequence comparison of viral isolates from those two tissues (brain or lymphoid/spleen) revealed five nucleotide changes but only two amino acid differences: one at residue 260 of GP1, phenylalanine (F) to leucine (L); and the other at residue 1079, lysine (K) to glutamine (Q) in the L protein [128,129,130,131] (Table 1A and B). For lymphoid isolates the predominant change was from phenylalanine to leucine (F260L) and the predicted GPC structures showed significant changes (Figure 3) that could affect GPC processing. 43 of the 47 spleen isolates (~91%) had L and 4 (~8%) had F. Meanwhile in variants selected in brain the F260 parental sequences were more abundant. 48 out of 59 had L260 (~81%) and 11 had F260 (~18%). There was no phenotype correlated with the RdRp K1079Q mutation alone [128,130,132]. However, another study showed that this mutation contributes to persistence and immunosuppressive phenotype [131]. Although the F260 mutation predominated in each tissue-specific isolate, there were still some F260 or L260 remaining quasi-species in each viral sample. Additionally, some viruses reverted to parental phenotype without changing back to the F260 residue [130]. Recent pyro-sequencing analysis of LCMV-Armstrong and -Clone 13 showed diversity in each viral preparation (Table 1A and B). The new sequencing of old isolates concluded that the original amino acid changes were indeed the major changes, with additional changes detectable but not dominant in the population. Researchers at Scripps and Geneva discovered an additional amino acid difference between LCMV isolates Clone 13 and Armstrong that were not corroborated by recent pyrosequencing studies of older isolates from the Salvato and Ahmed laboratories; however, reverse genetic studies found that the new amino acid changes did not affect the Clone 13 phenotype of persistence and immunosuppression [131,133]. The Clone 13 phenotype depended on contributions from both of the major mutations in the virus stock (the mutation in GP favored entry into dendritic cells and the mutation in the polymerase favored replication in dendritic cells resulting in increased antigen presentation and death of virus-specific T cells) [131,133]. 

Table 1LCMV sequence diversity. Comparison between different isolates of LCMV-Armstrong and LCMV-Clone 13. (**A**) Nucleotide and protein single nucleotide polymorphisms (SNPs) from the S segment. (**B**) Nucleotide and protein SNPs from the L segment. Black color letters represent the consensus sequence. Green, blue, and red letters represent abundance of that mutation at nearly 20%, 40% and 100%, respectively. Blue dark squares are the reported differences between both virus strains as follows: In the S segment nucleotide mutation 603 corresponds to amino acid 177, nucleotide mutation 855 corresponds to amino acid 260, nucleotide mutation 1298 corresponds to amino acid 407; in the L segment nucleotide mutation 3797 corresponds to amino acid 1079, nucleotide mutation 1798 corresponds to amino acid 412. The light blue square holds the previously reported mutation and confirmation of its presence in our laboratory strain. The Salvato 2012 pyro-sequencing shows diversity in those viral clones even within the same clone from the same laboratory.

**Table:** (**A**) S segmet

	S segment						
LCMV strains	UTR	GP1			GP2		NP
	nt/aa	nt/aa	nt/aa	nt/aa	nt/aa	nt/aa	nt
5’ end	467	603	606-7	855	1015-16	1298	2290
LCMV Armstrong	T/N	A/N	GC/A	T/F	AA/E	T/D	G/N
Grande-Perez *et al.* [101]							
LCMV Armstrong	C/N	G/D	CG/R	T/F	CC/A	T/D	G/N
Salvato *et al.* [17,129]							
LCMV Armstrong	C/N	A/D	GC/D	T/F	AA/E	T/D	A/N
Zapata *et al.* [135]							
LCMV C13	C/N	G/D	GC/D	**C/L**	AA/E	**C/D**	A/N
Flatz *et al.* [134]							
LCMV Cl 13	C/N	G/D	GC/D	**C/L**	AA/E	**C/D**	G/N
Zapata *et al.* [135]

**Table:** (**B**) L Segment

	**L Segment**															
LCMV strains	**IGR**			**L protein**											**UTR**	
	nt	nt	nt	nt/aa	nt/aa	nt/aa	nt/aa	nt/aa	nt/aa	nt/aa	nt/aa	nt/aa	nt/aa	nt/aa	nt	aa
5’ end	415	447	453	884	886	1201	1435	1798	2491	3797	5100	5795	7116	7165	7197	7200
LCMV Armstrong	G	G	G	A/T	T/T	T/H	G/V	G/A	C/F	A/K	A/K	T/L	C/T	G/L	T	C
Grande-Perez *et al.* [101]																
LCMV Armstrong	G	G		T/S	A/T	C/H	C/V	G/A	T/F	A/K	C/T	C/L	T/I	A/L	-	G
Salvato *et al.* [17,129]																
LCMV Armstrong	G	G	G	A/T	T/T	C/H	C/V	G/A	T/F	A/K	A/K	C/L	C/T	G/L	-	G
Zapata *et al.* [135]																
LCMV C13	A	G	G	A/T	T/T	C/H	C/V	**A**/A	T/F	**C/Q**	A/K	C/L	C/T	G/L	-	G
Flatz *et al.* [134]																
LCMV Cl 13	A	C	A	A/T	T/T	C/H	C/V	**A/A**	T/F	**C/Q**	A/K	C/L	C/T	G/L	-	G
Zapata *et al.* [135]																

In the Oldstone laboratory, a stock of the LCMV-WE strain was found to harbor viruses that did or did not cause growth hormone deficiency syndrome (GHDS) in persistently-infected C3H/st mice. A close analysis of plaque-purified viral clones illustrated that the virus strain contained variants of different phenotype. Less than 5% of the viral isolates were able to replicate to high titer in GH-producing cells and cause GHDS in C3H/st mice. The GHDS phenotype was due to a G to A nucleotide substitution at position 535 (serine 153 to phenylalanine at the protein level) of the LCMV-WE GP. However, the stable presence of GHDS-phenotypically silent variants in the LCMV-WE viral inoculum was not enough to induce disease, indicating that pathogenic variants can be maintained within a non-pathogenic viral population. Experimental mixtures of LCMV WEc54 (serine) and WEc2.5 (phenylalanine) showed that the proportion of the latter has to be between 10% and 50% in order to induce GHDS. In addition, this study found variability even inside each cloned virus [92,94].

In order to evaluate the role of the immune system in selecting variants of LCMV, different isolates from tissues of immunocompetent and immunocompromised animals were tested *in vivo*. After inoculation of adult mice, all animals showed the original pathogenic pattern. Viruses from lymphoid organs (Clone13-like) caused persistent infections and immunosuppression, in contrast to virus isolated from CNS. Those findings suggested that the immune system is not the main selective force and sequence analysis showed again that the mutation in the position 260 was tissue specific (F260L or F260I associated with lymphoid organs). Additionally, LCMV-Clone13-like viruses were found only in cells in the marginal zone and white pulp of the spleen, specifically in CD11c (+) and DEC-205 (+) splenic dendritic cells; while LCMV-Armstrong-like were found in the red pulp in CD11c (-) and DEC-205 (-) splenic dendritic cells. The specific tropism of those mutants was associated with increased receptor GP1/α–DG affinity. LCMV- Clone 13 (F260L or F260I) and LCMV WEc54 (S153) showed around 2–3 logs higher affinity for α–DG than their parental virus LCMV ARM and LCMV WEc2.2 (S153F) [132,136]. Those results indicated that receptor-virus interaction in target cells are selecting viral clones *in vivo*, defining the disease outcome. Additional selective pressures include enzyme processing rates, different nucleotide concentrations, codon bias, dose and route of infection and other microbes in the host. For example, there is some contribution from the arenavirus polymerase to the induction of persistence by LCMV-Clone 13, in that its mutation K1079Q gives it preferential ability to replicate in plasmacytoid dendritic cells over LCMV-Armstrong [131].

In conclusion, the analysis of specific mutations focuses on the function of each viral protein or genome component and its biological significance. However, the analysis of complex viral populations is in its infancy, and it could give a broader picture of how mixtures of particles, or quasi-species, impact pathogenesis and immunity. Therefore, the use of new tools such as deep sequencing and mathematical models can approach the pharmacological and vaccine research from a more holistic angle.

### 2.3. Arenavirus Variation after Passage in Multiple Hosts

In addition to their natural hosts, arenaviruses can infect other animals including hamsters, rabbits, squirrels, guinea pigs, dogs, chickens, bats, and primates. Some of them are refractory to disease [14,86,137,138,139,140,141,142,143,144]. Arenaviruses co-evolved with their natural host but little is known about those processes in other animals and the implication for pathogenesis. It has been demonstrated that the same virus induces different outcomes depending on the host. For instance, C3H/st, BALB/WEHI, and SWR/J mice infected at birth with LCMV-Armstrong, E-350, or Pasteur strains develop persistent infection but only C3H/st mice develop Growth Hormone Deficiency Syndrome. In contrast, LCMV strains Traub and WE failed to induce disease in those animals [145]. Examples can also be taken from the South American Guanarito virus (GTOV). GTOV virus isolated from rodents in Venezuela, showed higher sequence variation than human isolates suggesting differential host-specific selection of GTOV strains [146].

**Figure 4:**
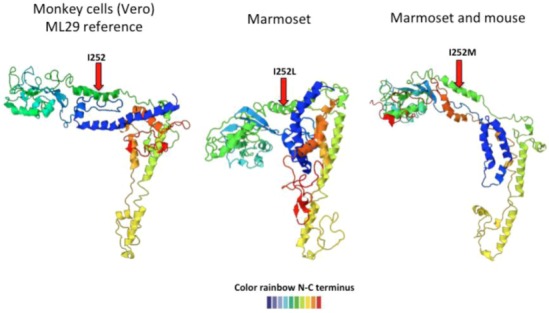
Predicted ML29 host-specific changes in GPC. The left-most structure shows the ML29 GPC predicted structure after passage in Vero cells. After inoculation into marmosets, the recovered viruses showed an isoleucine (I) to leucine (L) change at position 252 that affects the predicted GPC structure (middle structure). Another mutation at the same position, I to M, also changed GPC structure (figure on the right). The structure goes from N-terminus (blue) to C-terminus (red).

**Figure 5:**
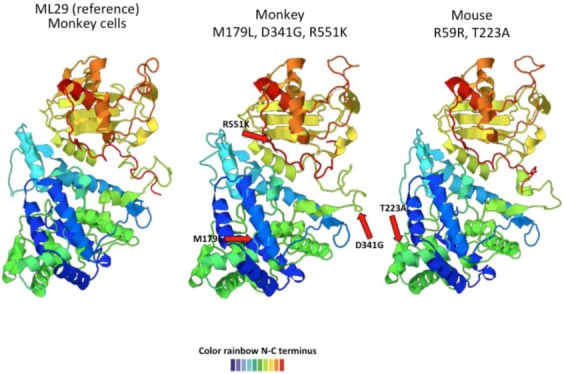
Predicted ML29 host-specific changes in NP. The left-most structure shows the ML29 NP predicted structure after passage in Vero cells. The middle structure represents the predicted NP changes (M179L, D341G, R551K) occurring in virus recovered from rhesus macaques only. The structure on the left shows two changes (R59R, and T223A) seen in mouse and marmosets. All host-specific mutations induced conformational changes in the NP predicted structure. The structure goes from N-terminus (blue) to C-terminus (red).

Since the Lassa vaccine candidate, ML29 was passaged in many different animals (mice, guinea pigs, marmosets, and rhesus macaques), and we took the opportunity to examine sequence variation with the goal of finding host-specific variants. In order to study the complexity and stability of ML29 *in vivo*, rhesus monkeys, marmosets, and mice were vaccinated and followed for several weeks by isolation of serum virus. Isolates were characterized by pyro-sequencing and showed several SNPs indicating the heterogeneity within cloned viral populations. As mentioned previously, the variation after 12 passages *in vitro* was less than 20% and within the expected mutation rates of arenaviruses. Some animals developed a very low viremia during the first two weeks after vaccination. ML29 virus isolated during these first few weeks was subjected to pyro-sequencing and proved to have a lower number of SNPs (less variation) than virus obtained from *in vitro* passage, and surprisingly, some of the variations were host-specific (Table 2). The virus-host adaptations also seemed to accumulate with time in the primate host [107]. Protein prediction analysis showed that those mutations, at the amino acid level, induced structural changes in GPC and NP (Figure 4 and Figure 5).

**Table 2:** Host-specific SNPs found after ML29 vaccination of different animals. Those specific mutations were found in rhesus macaque monkeys (Blue shade), marmosets (Pink shade), mouse (Orange shade) or common to marmoset and mouse (Green shade). Letters and numbers represent the amino acid change and the protein position. Those sites with the same amino acid represent synonymous changes.

Animal	Glycoprotein				Nucleoprotein	RdRp
Monkey	I 252				M 179 L	L 266 L
					D 341 G	L 494 L
					R 551 K	H 1572 Y
				
Marmoset		I 252 L	,	I 252 M		
					
Mouse	I 252 M				R 59 R	
					T 223 A	

Viral infection starts with binding to cellular receptors on the host cell. The virus:receptor interaction plays a role in tropism, host-range and pathogenesis. As discussed above, one amino acid change in the arenaviral GP or RdRp gives the virus the capacity to infect different cells and alters viral pathogenicity [128,130,147]. On the other hand, arenavirus receptors are highly conserved in vertebrates [148], though not all infected animals become productively infected. In fact, not all cells in the same individual expressing those receptors are infected, suggesting that differential receptor-processing, viral diversity, or other cellular factors are involved in viral tropism and virulence [148,149]. For instance, SKI-1/S1P cleavage of GPC from different OW arenaviruses, occurs at different rates suggesting that this protein acts as a selective factor, shaping the specific co-evolution of the arenaviruses and their hosts [150]. Additionally, GP, RdRp and NP sequences from LCMV-Armstrong, LCMV-C13, LASV, and ML29 showed synonymous mutations [128,129,130] (Table 1A and B) that could be also be giving advantage to some viral particles either by allowing the virus to use cells with specific pools of tRNA [151] or by changing the viral RNA structure, therefore changing the viral fitness and the virus phenotype. Evidence that synonymous mutations play a role in viral selection has been seen in long term HIV infection in which the viral population evolves towards the usage of host-preferred codons [152]. This is a field that needs further exploration and arenaviruses are suitable for such studies.

### 2.4. Examples of Arenavirus Variation due to Co-Evolution with other Viruses

**Figure 6:**
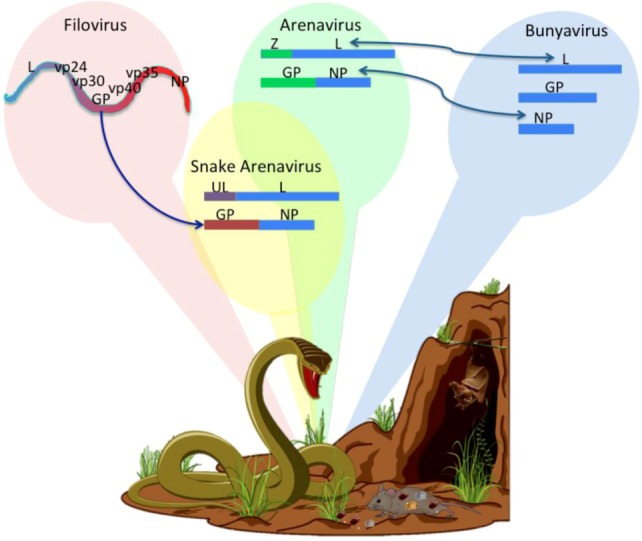
**Arenavirus Evolution.** The upper diagram shows the genomic structure and proteins encoded by filoviruses, arenaviruses, bunyaviruses, and the newly-discovered snake “arenavirus”. The blue arrows show the hypothetical changes that occurred in the arenaviral ancestors. The new snake virus has the L and NP genes of an arenavirus, but the GP of a filovirus and the Z gene similar to a cellular ubiquitin ligase (UL). The lower diagram shows how the reservoirs of arenaviruses, filoviruses, and bunyaviruses can interact with each other in the same ecological niche allowing co-infections and mixing of viral genomes to produce new viruses (Modify from [159]).

Sequence analysis of recently emerging viruses suggested that genes from arenaviruses share some homology with other negative-strand virus such as filoviruses and bunyaviruses [153,154]. The tick-borne Crimean Congo Hemorrhagic Fever Virus (CHFV) is a bunyavirus, genus Nairovirus, with three genomic RNA segments: the largest encodes a polymerase (with strong homology to the Lassa L RdRp), the middle segment encodes a bunyavirus-like envelope glycoprotein Gc,Gn, and the smallest segment encodes a nucleocapsid protein with strong homology to the Lassa NP [154]. It is possible that arenaviruses and bunyaviruses shared an ancestor. For example, a precursor of CCHFV could have adapted to propagate in both insects and mammals and could have fragmented a genome segment to evolve from 2 to 3 segments. One can also imagine that a virus like CCHFV initiated during a co-infection of ticks or mammalian hosts with a bi-segmented arenavirus and a tri-segmented bunyavirus. Co-infection could enable the bunyavirus polymerase to jump templates from a bunyaviral replicative form to an arenaviral mRNA (Figure 6). Mixing of these viruses is helped by the fact that between 4%–30% of bunyaviruses package two genome equivalents per virion [155,156,157]. 

A new type of bi-segmented negative-strand RNA virus has recently been discovered in snakes. Three isolates of the new virus are characterized by ambisense coding and some sequence-homology with arenaviruses. The snake virus L and NP genes are homologous to those of arenaviruses, the GP sequences are homologous to filovirus envelope glycoproteins, and the Z gene sequences are homologous to host ubiquitin ligase [158]. Since the new virus was discovered in snakes suffering from Inclusion Body Disease (IBD) characterized by a build-up of virus particles in cellular cytoplasm, we speculate that the new virus carries a defective Z protein that is inefficient in packaging, exit, and transmissibility but better suited for chronic infection, a characteristic that would favor virus:host co-adaptation. Perhaps such viruses are only recently acquired by snakes since IBD is a lethal disease. Some snakes consume rodents and bats that carry arenaviruses and filoviruses; so a co-infected snake could conceivably harbor replication complexes in which the arenavirus polymerase jumps from an arenaviral replicative form to a filovirus GP mRNA or to a snake endogenous ubiquitin ligase (Figure 6). The snake viral Z protein could also be a primordial Z protein derived from a host ubiquitin ligase that functions perfectly well in budding. It would be highly interesting to substitute the new Z protein into functional-budding assays, or to monitor viral co-infection in snake cells to test the feasibility of recombination events.

Another possible source of arenavirus variation comes from co-infection with retroviruses or infection in the presence of a reverse transcriptase/retrotransposase. This could result in the integration of arenavirus sequences into the somatic genomic material of a host organism. The fact that this phenomenon takes place has resulted in speculation about its effect on low-level arenaviral gene expression leading to self-tolerance of arenaviral antigens [160,161].

Outside the laboratory, the real world is a very complicated microbiome, and the few examples of co-mingling of arenavirus sequences and host or other microbe sequences broadens the possibilities for arenavirus evolution.

## 3. Conclusion

In conclusion, viral genetic diversity plays an important role in the viral population fitness that is shaped by different selective pressures in the host, determining the host range of the viral offspring and the outcome of the infection. Therefore, it is important to study the heterogeneity and complexity of viral populations in the context of viral evolution and pathogenicity. Such observations will constitute a model for developing better viral classification systems, better vaccine candidates or vaccine screening techniques, and new drugs for viral treatments. 

## Appendix

**Supplementary Table 1:** List of all isolated arenavirus and their name, year of isolation, geographic distribution, reservoir hosts, and associated human diseases. The arenaviruses are divided into two serocomplexes: Old World and New World and based on the viral nucleocapsid protein gene sequences the NW viruses are divided into three phylogenetic groups

Virus Species	Origen of name	Year	Distribution	Host	Associated Disease
Old World or LASV-LCMV serocomplex					
Lymphocytic choriomeningitis virus (LCMV)	Disease	1933 [162]	Worldwide	House mouse *(Mus musculus)* and (*Mus domesticus*) and Syrian hamster (*Mesocricetus auratus*)	Flu like symptoms Meningitis
					Encephalitis
					Congenital abnormalities/Abortion
					Multisystem organ failure in transplanted patients
Lassa (LASV)	Town, Nigeria	1969 [163]	West Africa	Multimammate mouse (*Mastomys* genus)	Hemorrhagic fever
Mopeia (MOPV)	Town, Mozambique	1977 [164]	Southern Africa	Multimammate mouse *(Mastomis natalensis)*	Not associated with human disease
Merino Walk	Farm, South Africa	1985 [55]	Eastern Cape	Karoo rat (*Myotomys unisulcatus*)	Unknown pathogenicity for humans
(MWV)			South Africa		
Mopeia/Lassa	M/L reassortant clone 29	1992 [72]	Russia	*Laboratory virus.* Passaged *in Vero E6 and BHK cells*	Vaccine candidate against LHF
reassortant					
(ML29)					
Morogoro	City, Tanzania	2009 [165]	East Africa	Multimammate mouse *(Mastomys natalensis)*	Unknown pathogenicity for humans
			Tanzania		
Mobala (MOBV)	Region, DR of Congo	1983 [166]	Central African Republic	Soft-furred rat (*Praomys* sp.)	Not associated with human disease
IPPY (IPPYV)	Town, Central African Republic	1985 [167]	Central African	Nile grass rat (*Arvicanthis* sp.)	Not associated with human disease
Lujo (LUJV)	Lusaka, Zambia Johannesburg, South Africa	2009 [54]	Southern Africa	Unknown	Hemorrhagic fever
Luna (LUNV)	Lusaka-Namwala, Zambia	2009 [168]	Southern Africa Zambia	Multimammate mouse *(Mastomys natalensis)*	Unknown pathogenicity for humans
**New World arenavirus or Tacaribe serocomplex South American group**					
**Group A**					
Pichindé (PICV)	Valley, Colombia	1965 [169]	South America	Tome’s rice rat (*Oryzomys albigularis*)	Not associated with human disease
			Colombia		
Paraná (PARV)	River, Paraguay, Brazil, Argentina	1965 [170]	South America	Paraguayan rice rat (*Oryzomys buccinatus, Oryzomys angouya*)	Not associated with human disease
			Paraguay		
Flexal (FLEV)	Brazil	1975 [171]	South America	Tome’s rice rat (*Oryzomys albigularis, Nephelomys albigularis*), Paraguayan rice rat (*Oryzomys angouya, Oryzomys buccinatus*),	Febril illness Associated with nonfatal laboratory-acquired infection
			Brazil		
Pirital (PIRV)	Community, Venezuela	1995 [172]	South America	Alston’s cotton rat (*Sigmodon alstoni*)	Not associated with human disease
			Venezuela		
Allpaahuayo (ALLV)	National reserve, Peru	1997 [173]	South America	*Arboreal rice rats (Oecomys bicolor and Oecomys paricola)*	Unknown pathogenicity for humans
			Peru		
**Group B**					
Tacaribe (TCRV)	Beach, Trinidad	1956 [174]	Caribbean Sea	fruit-eating bat (*Artibeus* sp.)	Associated only with single, nonfatal, laboratory-acquired infection.
			Trinidad		
Junin (JUNV)	Town, Argentina	1958 [175]	South America	Corn mouse, drylands vesper mouse *(Calomys masculinus*), grass field mouse *(Akodon azarae*), dark field mouse *(Bolomys obscurus*)	Hemorrhagic fever
			Argentina		
Candid#1	Argentina	1985 [176]	South America	Passaged guinea pigs (GP2), mouse	Vaccine against Argentinian hemorrhagic fever
			Argentina	(MB44), followed by clonal selection	
				in fetal rhesus monkey lung cells (FRhL19).	
Machupo (MACV)	River, Bolivia	1962 [28]	South America	large vesper mouse *(Calomys callosus*)	Hemorrhagic fever
			Bolivia		
Amapari (AMAV)	Amapá region, Brazil	1964 [177]	South America	rice rat *(Oryzomys goeldii*), bristly mouse *(Neacomys guianae*)	Not associated with human disease
			Brazil		
Cupixi (CPXV)	Town, Brazil	1970 [77]	South America	Large-headed Rice Rat *(Oryzomys capito)*	Not associated with human disease
			Brazil		
Guanarito (GTOV)	Region, Venezuela	1989 [178]	South America	Cane mouse *(Zygodontomys brevicauda*)	Hemorrhagic fever
			Venezuela		
Sabiá (SABV)	Town, Brazil	1990 [179]	South America	Unknown (Suspected rodent)	Hemorrhagic fever, Hemorrhagic fever associated with nonfatal laboratory-acquired infection
			Brazil		
Chapare (CHPV)	Town, Bolivia	2005 [180]	South America	Unknown	Hemorrhagic fever
			Bolivia		
**Group C**					
Latino (LATV)	Bolivia	1965 [31]	South America	Large vesper mouse (*Calomys callosus*)	Not associated with human disease
			Bolivia, Brazil		
Oliveros (OLVV)	Town, Argentina	1990 [181]	South America	Dark bolo mouse (*Bolomys obscurus*)	Not associated with human disease
			Argentina		
Pampa virus (PAMV)	Region, Argentina	1997 [182]	South America	Dark bolo mouse (*Bolomys sp.*)	Not associated with human disease
			Argentina		
**North American group**					
**Group A**					
Tamiami (TAMV)*	Everglades, USA	1964 [183]	North America Florida	Hispid cotton rat *(Sigmodon hispidus*)	Not associated with human disease
Whitewater Arroyo* (WWAV)	Whitewater Creek	1993 [184]	North America New Mexico	White-throated wood rat *(Neotoma albigula*)	Febrile infection
					Respiratory distress syndrome
Catarina (CTNV)	Town, USA	1999 [185]	North America Texas	Southern Plains Woodrat (*Neotoma micropus*)	Unknown Pathogenicity for humans
Skinner Tank virus	Reservoir, USA	2002 [186]	North America Arizona	Mexican woodrat *(Neotoma Mexicana*)	Unknown pathogenicity for humans
(SKTV)					
Big Brushy Tank	USA	2008 [187]	North America Arizona	White-throated woodrat (*Neotoma albigula*)	Unknown pathogenicity for humans
(BBTV)					
Tonto Creek	Creek, USA	2008 [187]	North America Arizona	White-throated woodrat (*Neotoma albigula*)	Unknown pathogenicity for humans
(TTCV)					
Bear Canyon (BCNV)*	Trailhead, USA	2002 [188]	North America California	California mouse *(Peromyscus californico*), Large-eared woodrat *(Neotoma macrotis*)	Unknown pathogenicity for humans

*****: represents recombinant viruses. [25]

### Supplementary part 2 - Genetically detected arenaviruses

#### Old World

Gbagroube virus and Menekre virus: Mus setulosus, mice. Unknown pathogenicity for humans. Côte d’Ivoire, 2005 [56].

Kodoko virus: Guinea. Nannomys minutoides, Mus (Nannomys). Unknown pathogenicity for humans. West Africa, 2007 [189].

Dandenong (DANV): Town, Australia. Unknown host. Eastern Europe. Encephalitic illness in transplanted patients, 2008 [190].

#### New World

Black Mesa virus: Similar to WWAV. Black mesa state park. Oklahoma. USA. Neotoma albigula (Rat). Unknown pathogenicity for humans, USA, 1985 [191].

Oro Grande virus: Community California. USA. Neotoma micropus (Rat). Unknown pathogenicity for humans, 1998 [192].

Rio Cacarana virus (RCAV): Argentina, 2006 [193].

Real de Catorce (RDCV): Real de Catorce locality. San Luis Potosí. White-toothed woodrat (*N. leucodon*). Unknown pathogenicity for humans, Mexico, 2010 [194].

Pinhal virus: Close to LATV and OLVV. Calomys tener (delicate vesper mouse). Unknown pathogenicity for humans, Brazil, 2007 [195].

Ocozocoautla de Espinosa virus: Municipality, Chiapas, Mexico. Mexican deer mice (*Peromyscus mexicanus*). Closely related to South American Tacaribe complex. Unknown pathogenicity for humans, Mexico, 2012 [196]
